# Ibuprofen gargle for quality of life and pain improvement in oral lichen planus: randomized crossover and long-term extension phase II study

**DOI:** 10.1186/s40780-025-00536-0

**Published:** 2026-01-08

**Authors:** Yumi Kitahiro, Yasumasa Kakei, Takeshi Ioroi, Nanae Yatagai, Masahiko Kashin, Masaki Kobayashi, Asami Morioka, Kazuhiro Yamamoto, Takumi Hasegawa, Masaya Akashi, Ikuko Yano

**Affiliations:** 1 Department of Pharmacy, Kobe University Hospital, 7-5-2 Kusunoki-cho, Kobe, Hyogo, 650-0017 Japan; 2 Department of Oral and Maxillofacial Surgery, Kobe University Graduate School of Medicine, 7-5-2 Kusunoki-cho, Kobe, Hyogo, 650-0017 Japan; 3 Department of Integrated Clinical and Basic Pharmaceutical Sciences, Faculty of Medicine, Dentistry and Pharmaceutical Sciences, Okayama University, Okayama, 700-8558 Japan

**Keywords:** Oral lichen planus, Ibuprofen gargle, PROMS, Oral pain, Long-term extension study

## Abstract

**Background:**

Oral lichen planus (OLP) is a chronic immune-mediated inflammatory disease of the oral mucosa that frequently causes erosive lesions accompanied by pain, thereby impairing the patient’s ability to eat and drink and their overall quality of life (QOL). Current guidelines recommend topical corticosteroids as first-line treatments; however, their efficacy is limited for pain relief, and long-term safety remains a concern. Nonsteroidal anti-inflammatory drug mouthrinse formulations can deliver high local drug concentrations to the symptomatic mucosa with minimal systemic exposure. This study evaluated the short- and long-term safety and efficacy of ibuprofen gargling in patients with painful OLP.

**Methods:**

We conducted a randomized, double-blind, placebo-controlled crossover study (Days 1–7), followed by a 6-month open-label long-term extension (LTE) study (Days 8–176). Patients with OLP and baseline oral pain of ≥ 20 mm on a 100-mm visual analog scale (VAS) were enrolled. The LTE study’s primary endpoint was safety, and secondary endpoints included changes in the VAS in resting oral pain and oral health-related QOL, which was measured using the Patient-Reported Oral Mucositis Symptom (PROMS) scale.

**Results:**

The crossover study enrolled 24 patients, and 18 patients continued the LTE study. No serious adverse events were observed. One patient discontinued treatment because of grade 2 oral pain, while the remaining patients tolerated long-term treatment exhibiting stable laboratory values. During the LTE study, ibuprofen gargling was associated with significant improvements in several PROMS domains. The most pronounced effects were observed in the dietary domains, including eating restriction (β_day_ = − 0.083, *p* < 0.001) and difficulty eating hard foods (β_day_ = − 0.067, *p* < 0.001). Modest but significant improvements were observed for mouth pain (β_day_ = − 0.038, *p* = 0.029), difficulty eating soft foods (β_day_ = − 0.021, *p* < 0.001), swallowing (β_day_ = − 0.007, *p* < 0.001), and drinking (β_day_ = − 0.006, *p* = 0.043).

**Conclusions:**

A 6-month regimen of ibuprofen gargling may be safe and associated with modest and gradual improvements in pain and oral function-related QOL in patients with OLP. Further confirmatory study is needed to confirm the potential therapeutic role of ibuprofen gargling.

**Trail registration:**

The Registry of Clinical Trials; jRCTs051220009 and jRCTs051220010, date of registration: 22 April 2022.

## Background

Oral lichen planus (OLP) is a chronic immune-mediated inflammatory disorder that affects the oral mucosa and frequently presents as erosive lesions, which are accompanied by pain. OLP adversely affects the patient’s oral function and quality of life (QOL), particularly with regard to eating and drinking [1, 2]. Current guidelines as well as systematic reviews recommend topical corticosteroids as the first-line symptomatic treatment; however, evidence supporting their pain reduction efficacy is limited, and concerns regarding their long-term tolerability, such as candidiasis or mucosal atrophy, remain prevalent in clinical practice [3].

Mouthrinse formulations can deliver high drug concentrations directly to the painful mucosal surfaces while minimizing systemic exposure to the drug. A previous study has demonstrated the feasibility of topical nonsteroidal anti-inflammatory drugs (NSAIDs) gargles, such as ibuprofen and diclofenac, for painful orofacial conditions, including treatment-related oral mucositis or postoperative periodontal pain [4]. Therefore, locally administered NSAIDs may be beneficial as adjuncts or alternatives to support OLP treatments. In particular, ibuprofen, as a non-selective cyclooxygenase inhibitor with well-established analgesic properties, is particularly promising for short-contact topical use, as a brief swish-and-spit regimen may provide rapid symptom relief with minimal systemic dose exposure. In a previous feasibility study, an ibuprofen gargle reduced mucositis-related pain within minutes and exhibited an acceptable safety profile, thus suggesting that a targeted oral mouthrinse formulation may be clinically beneficial despite potential local irritation [4].

Measures of patient-reported outcomes that reflect mouth-related function are essential to evaluate the meaningful benefits of OLP treatments [5]. The Patient-Reported Oral Mucositis Symptom (PROMS) scale is a validated tool that captures various domains, such as mouth pain, eating and drinking difficulties, and speech, which closely align with the symptomatic burden of erosive oral diseases such as OLP [6]. Furthermore, its measurement properties and clinical interpretability support its use as a practical QOL endpoint for interventional studies.

Therefore, we designed a placebo-controlled, double-blind, randomized crossover study (Days 1–7) that was followed by a long-term extension (LTE) study (Days 8–176) to evaluate the short-term analgesic effects and the long-term patient-centered outcomes of ibuprofen mouthrinse gargling for OLP lesion-related pain [7]. The study protocol prespecified a change in pain based on a visual analog scale (VAS) at 5 min after gargling as the primary endpoint of the crossover phase. There was no significant difference in the degree of pain reduction in the VAS values between the ibuprofen and placebo groups before and 5 min after gargling. However, as the secondary endpoint, the PROMS scale values revealed a significant reduction in the dietary domain restrictions (*p* = 0.032) in favor of the ibuprofen gargle compared to that of the baseline [8]. In the LTE study, the primary endpoint was the safety assessment of the ibuprofen gargle, which was evaluated according to the Common Terminology Criteria for Adverse Events (CTCAE) version 5.0. Furthermore, the secondary endpoints were to evaluate the PROMS assessments to characterize the trajectories of the patients’ mouth-related QOL. In this report, we focused on the integrated outcomes of the randomized crossover and LTE studies, which included (i) the long-term safety of ibuprofen gargling, (ii) the long-term changes in pain perception using ibuprofen gargling, and (iii) the long-term changes across the various PROMS domains.

## Methods

### Study design

This study was designed as a placebo-controlled, double-blind, randomized crossover study (Days 1–7) followed by an open-label LTE study (Days 8–176). The study aligned with the CONSORT 2010 statement guidelines [9].

### Inclusion and exclusion criteria

In the randomized crossover study, individuals aged ≥ 20 years at the time of consent, diagnosed with OLP, and with an oral lesion averaging ≥ 20 mm on a 100-mm VAS during 7 days before enrollment were included. Patients were excluded from the study for the following reasons: peptic ulcer disease, uncontrolled comorbidities, hypersensitivity to ibuprofen or its excipients, clinically important cardiac disease, aspirin-induced asthma, frequent use of systemic analgesics for chronic pain, pregnancy or lactation, neuropsychiatric conditions that precluded compliance with study procedures, and other concerns determined by the investigator as valid cause for exclusion. Ongoing local and systemic therapies for oral lesions were permitted if the dose was stable for ≥ 28 days prior to enrollment. In the LTE study, the eligibility criteria included patients who had completed the crossover study and wished to continue using the ibuprofen gargle. The exclusion criteria included patients who met the termination criteria within 2 days of the crossover study start.

### Interventions

The study drug was prepared by the Department of Pharmacy at Kobe University Hospital. Furthermore, for the short-term crossover study, to maintain investigator blinding, a non-blinded pharmacist prepared the study medication.

For the randomized crossover study (Days 1–7), patients were randomly assigned to receive either “ibuprofen followed by placebo” or “placebo followed by ibuprofen” during the first two days, with both groups then receiving ibuprofen gargle on Days 3–5 [7]. During the LTE study (Days 8–176), all patients received ibuprofen gargle every day. They were instructed to use the gargle at least once a day. Moreover, the patients were allowed to initiate new treatments for oral lesions, thereby reflecting clinical conditions during the LTE study.

### Endpoints

The primary endpoint of the LTE study was the evaluation of adverse events according to the CTCAE version 5.0. The secondary endpoints included the gargle’s effectiveness based on changes in the VAS for resting oral pain from the baseline (i.e., before gargling) and oral health-related QOL improvements, as measured by the various PROMS domains. In this report, we present the integrated outcomes of the randomized crossover and LTE studies, with a particular focus on long-term pain perception and long-term changes across the various PROMS domains, compared with using ibuprofen gargle on Day 1.

### Assessments

#### Safety assessment

Adverse events related to the study drug were assessed by the investigator. In addition, follow-up visits were scheduled every 56 days (Days 64, 120, and 176) with an allowable visit window of ± 14 days.

#### Evaluation of the long-term efficacy of the ibuprofen gargle

Patients reported the severity of pain using the VAS, ranging from 0 mm (no pain) to 100 mm (worst pain), which was recorded immediately before as well as 5 and 15 min after gargling every 7 days. These endpoint times were based on a previous study, which investigated the efficacy of ibuprofen gargles for oral mucositis [10, 11].

#### QOL evaluation using the various PROMS domains

Patients each assessed their QOL using the various PROMS domains from 0 mm (no problem) to 100 mm (worst difficulty), and this was recorded every 7 days. The prespecified PROMS domains included mouth pain; difficulty in speaking, drinking, swallowing, and eating soft and hard foods; and restriction of speech, eating, and taste.

### Statistical analysis

The safety data, including all treatment-related adverse events, were summarized as counts. The effectiveness set included patients in the LTE study with at least one post-baseline PROMS assessment. The safety set included patients who used the ibuprofen gargle at least once during the LTE study. All statistical tests were two-sided, with α = 0.05. The longitudinal changes in the various PROMS domains and VAS scores were analyzed using linear mixed-effects models (LMMs). These models included a fixed effect for day (as a continuous variable) and a random intercept for each patient to account for repeated measurements. This was represented as value ~ day + (1/subject) in the analysis code. The degrees of freedom were calculated according to the Kenward-Roger approximation. The model-estimated slope (β_day_​) was used to quantify the average daily change for each outcome, and the intraclass correlation coefficient (ICC) was also calculated from the same models to summarize between- and within-subject variability. These analyses were performed using R software (R Foundation for Statistical Computing, version 4.2.1).

## Results

### Patient characteristics

**Fig. 1:**
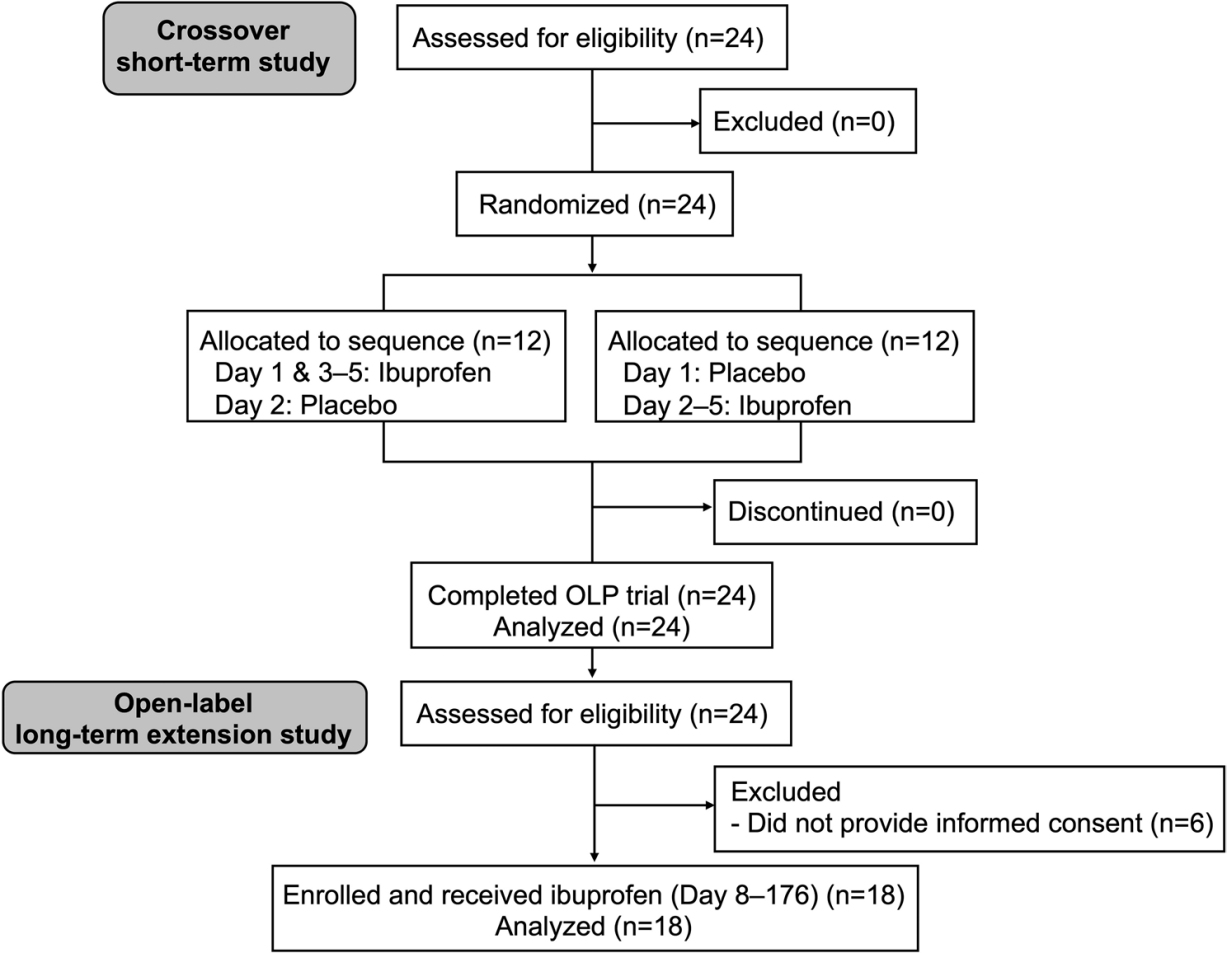
CONSORT flowchart showing the study design. OLP: oral lichen planus

Figure 1 shows the CONSORT flowchart of the study. In the initial randomized crossover study, a total of 24 patients signed an informed consent form between June 2, 2022, and July 11, 2024. Thereafter, in the follow-up LTE study, 18 patients were included between June 23, 2022, and July 19, 2024. The baseline characteristics of the patients are presented in Table 1.

**Table 1:** Patient characteristics

	*N* = 18
Age, years^1^	60 (36 − 85)
Sex, female, n (%)	15 (83.3)
Duration of OLP, month^1^	24 (2 − 180)
Lesion site, n	
Number of lesion sites per patient^1^	2 (1 − 4)
Buccal mucosa (total number of right and left)	21
Maxillary gingiva (total number of right and left)	5
Mandibular gingiva (total number of right and left)	6
Tongue (total number of right and left)	0
Ongoing treatment for oral lesions at baseline, n	
Azunol Gargle liquid	1
Dexamethasone Ointment	1
Azunol Gargle liquid and Dexamethasone Ointment	2

^1^Data are presented as the median (range)

Some patients had multiple lesions at a single site; therefore, the total number of buccal mucosa lesions exceeds the total number of patients (*N* = 18)

OLP: oral lichen planus

### Safety assessment

No serious adverse events were observed during the LTE study. However, one patient experienced grade 2 oral pain on Day 9, which was considered a treatment-related adverse event, and this event resolved after the patient discontinued study treatment. No other patients discontinued the study due to adverse events. Laboratory data, including renal and liver function values, remained stable throughout the study, and no clinical changes were detected between Days 8 and 176 (Table 2).

**Table 2:** Laboratory data

	Day 8 (*N* = 18)	Day 176 (*N* = 14)
WBC (cells/µL)	6,400 (3,700 − 10,800)	5,150 (3,900 − 8,500)
Plt (10^4^/µL)	22.1 (8.3 − 32.2)	25.5 (7.7 − 36.3)
Hb (g/dL)	13.3 (10.6 − 16.3)	13.3 (9.9 − 16.4)
AST (U/L)	21 (10 − 72)	20 (16 − 76)
ALT (U/L)	17 (10 − 96)	19 (10 − 62)
γ-GTP (U/L)	18 (12 − 223)	24 (12 − 324)
T-Bil (mg/dL)	0.7 (0.4 − 1.1)	0.6 (0.4 − 1.3)
Alb (g/dL)	4.2 (3.0 − 4.6)	4.1 (3.3 − 4.6)
SCr (mg/dL)	0.64 (0.41 − 0.98)	0.63 (0.47 − 0.94)
BUN (mg/dL)	13.4 (7.3 − 26.3)	13.3 (8.2 − 22.1)

All data are presented as the median (range). The post-laboratory data on Day 176 were obtained with an allowable visit window of ± 14 days. Alb: albumin, ALT: alanine aminotransferase, AST: aspartate aminotransferase, BUN: blood urea nitrogen, γ-GTP: γ-glutamyl transpeptidase, Hb: hemoglobin, Plt: platelet count, SCr: serum creatinine, T-Bil: total bilirubin, WBC: white blood cell count

### Concomitant medications

At baseline, 4 of 18 patients used concomitant topical medications for oral lesions (Table 1). One of these patients withdrew early due to a treatment-related adverse event on Day 9, and three patients withdrew during follow-up; therefore, paired evaluation was performed among the 14 patients with available data at both baseline and the end of the LTE period. Among these 14 patients, concomitant topical medications were used by 3 patients at baseline and by 3 patients at the end of the LTE period; no patient initiated or discontinued these medications (initiation 0, discontinuation 0; exact McNemar test, *p* = 1.00). No concomitant systemic analgesics were recorded during the LTE study.

### Long-term effectiveness of the ibuprofen gargle

#### Resting oral pain

**Fig. 2:**
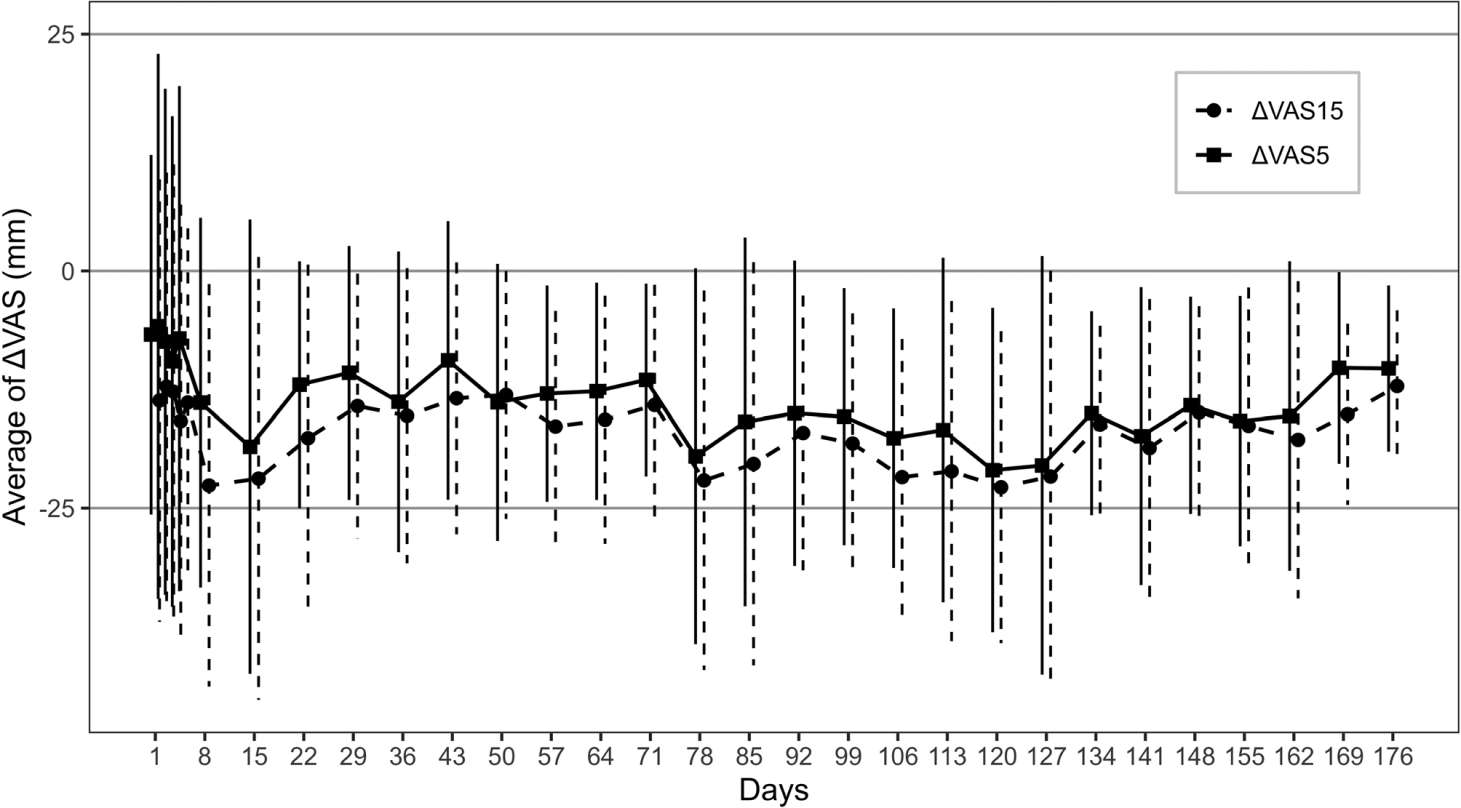
Mean change in the pain VAS at 5 and 15 min post-gargling. Vertical means standard deviation. VAS: visual analogue scale

**Fig. 3:**
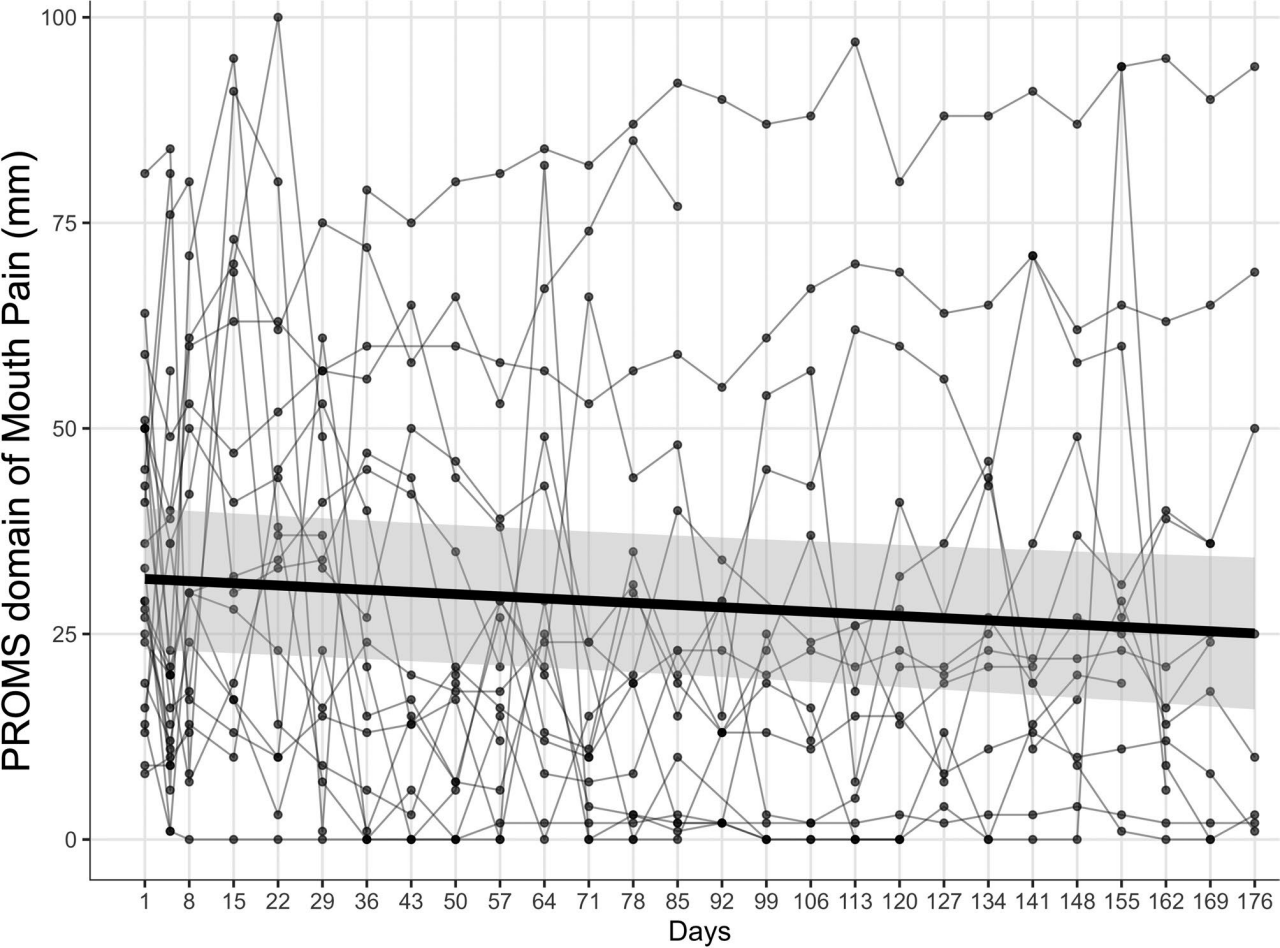
Individual and population-level trajectories for the PROMS domain of mouth pain. The black line represents the mean of the population. The shaded area represents the 95% confidence interval of the population-level trajectory estimated from the linear mixed-effects model. PROMS: patient-reported oral mucositis symptom

The mean change in the pain VAS at 5 and 15 min post-gargling is presented in Fig. 2, which demonstrates the long-term analgesic effect of the ibuprofen gargle. The longitudinal trajectory of resting (pre-gargle) oral pain during the LTE study mirrored the improvements observed in the mouth pain PROMS domain. As shown in Fig. 3, which plots individual and population-level trajectories for mouth pain and is most closely associated with the change in oral pain VAS, the mouth pain PROMS domain scores demonstrated a modest but significant improvement over the 176 days of observation. The LMMs estimated a significant mean decrease per day (β_day​_ = −0.038, 95% Confidence Interval (CI): −0.072 to − 0.004; *p* = 0.029). The between-subject variance of the random intercept was 349 (Standard Deviation; SD 18.7) and the residual variance was 305 (SD 17.5), yielding an ICC of 0.53.

#### PROMS domains

Significant improvements were observed across various other PROMS domains (Table 3). The most pronounced effects were associated with diet, including restriction of eating (β_day_​ = −0.083, *p* < 0.001) and difficulty eating hard foods (β_day​_ = −0.067, *p* < 0.001). In addition, significant improvements were found for difficulty eating soft foods (β_day_​ = −0.021, *p* < 0.001), difficulty swallowing (β_day_​ = −0.007, *p* < 0.001), and difficulty drinking (β_day_​ = −0.006, *p* = 0.043).

**Table 3:** Linear mixed-effects estimates of time trends in each Patient-Reported oral mucositis symptom (PROMS) domain (Days 1–176)

PROMS domain	β_day_	SE	95% CI	*p*-*value*	ICC
Mouth pain	−0.038	0.017	−0.072 to − 0.004	0.029	0.53
Difficulty speaking	−0.017	0.009	−0.035 to 0.000	0.054	0.66
Restriction of speech	−0.006	0.008	−0.021 to 0.010	0.479	0.71
Difficulty eating hard foods	−0.067	0.015	−0.096 to − 0.038	< 0.001	0.75
Difficulty eating soft foods	−0.021	0.005	−0.031 to − 0.010	< 0.001	0.35
Restriction of eating	−0.083	0.012	−0.107 to − 0.059	< 0.001	0.66
Difficulty drinking	−0.006	0.003	−0.012 to − 0.000	0.043	0.22
Restriction of drinking	−0.007	0.004	−0.015 to 0.001	0.090	0.78
Difficulty swallowing	−0.007	0.002	−0.011 to − 0.003	< 0.001	0.56
Change in taste	0.017	0.009	−0.001 to 0.035	0.064	0.23

For each domain, β_day_ is the estimated mean daily change in the score from a model with a fixed effect for day and random intercepts by individual patient (*value* ~ *day* + (1|Subject)). Days 1–7 correspond to the randomized crossover phase; Days 8–176 correspond to the long-term extension (LTE), aligned by adding 7 to the LTE days. The intraclass correlation coefficient (ICC) was calculated from the same model to summarize within- and between-subject variability in scores. Entries are the estimate (β_day_), standard error (SE), 95% confidence interval (CI), two-sided *p-value*, and intraclass correlation coefficient (ICC). Negative β_day_ indicates decreasing domain scores (i.e., improvement). No adjustment for multiple testing was applied

## Discussion

To the best of our knowledge, this is the first long-term phase II study to evaluate the safety and efficacy of ibuprofen mouthrinse gargling in patients with OLP. Previous studies have primarily focused on short-term symptomatic relief or on the use of topical corticosteroids [12], rather than on NSAIDs gargles over extended periods. Our study revealed a favorable safety profile and significant improvements in patient-reported outcomes, particularly with regard to oral function, such as eating and drinking. A previous study reported that betamethasone mouthwash exhibited efficacy in rapidly enhancing erosion healing within 2 weeks and extending the recurrence interval with a good safety profile [13]. The effectiveness of ibuprofen gargling appeared to be lower than that of corticosteroid gargling. Therefore, our findings highlight the potential of locally administered NSAIDs as adjunctive therapeutic options for patients with OLP; cannot tolerate corticosteroids due to candidiasis or other adverse effects, require long-term symptom management where corticosteroid-sparing strategies are desired, and have predominantly symptomatic complaints without severe erosive disease, a condition that remains challenging to manage with currently available treatments.

The most clinically relevant observation was the sustained improvement in the dietary-related PROMS domains, which included restrictions on eating and difficulty in consuming hard and soft food. As OLP pain often manifests most severely during mastication, improvements in these domains are a direct reflection of the functional benefits in the patients’ daily QOL. In contrast to the immediate but modest analgesic effect observed in the crossover phase, results of the long-term study suggest that continued use of the ibuprofen gargle provides incremental and cumulative benefits, not only in reducing resting oral pain but also in alleviating functional impairment in essential activities, such as eating, drinking, and swallowing. These improvements are of particular importance because food-related QOL is consistently reported as one of the most burdensome aspects of OLP. A previous study reported that over 90% of patients with OLP reported discomfort with specific types of food, and many reported limiting the texture and types of food that they consumed [14]. Effects such as difficulty eating certain foods, which can lead to weight loss or, in severe cases, malnutrition, have been reported. Therefore, dietary satisfaction is a risk that can influence patients’ happiness and social abilities [15, 16].

Another positive advantage of ibuprofen gargling is its safety profile. The targeted swish-and-spit administration allows high local ibuprofen concentrations while minimizing the systemic absorption thereof, thereby reducing the risks associated with long-term NSAIDs use, including gastrointestinal bleeding, renal impairment, or cardiovascular complications. Furthermore, after six months of daily use, no serious adverse events were observed, and laboratory data findings remained stable, thus reinforcing the tolerability of this approach in a chronic disease population. Although OLP lesions were clinically assessed at each visit, we did not prospectively grade lesion stage or quantify lesion size; therefore, our study cannot directly evaluate whether ibuprofen gargling delayed mucosal healing. A Previous study reported that oral ibuprofen can delay the healing of extraction sockets [17], it cannot be ruled out that long-term use of ibuprofen gargles may be worsening the condition. In contrast, a previous report suggested that low-dose NSAIDs do not appear to have a detrimental effect on soft tissue healing, although possible effects on bone healing have been discussed [18]. Additionally, randomized controlled trials of a low-dose ibuprofen-releasing foam dressing (Biatain-Ibu) in patients with venous leg ulcers, have reported that reduction in ulcer area was comparable between ibuprofen and non-ibuprofen foam dressings [19, 20]. Although these data are derived from cutaneous wounds rather than oral mucosa, they suggest that short-term, low-dose local ibuprofen is unlikely to have a major adverse effect on tissue repair; nevertheless, prospective evaluation of OLP lesion severity in future studies is warranted.

This study has several limitations that should be acknowledged. First, the LTE phase was performed in an open-label manner without a placebo control, which potentially introduced placebo effects and observer bias. Although patients served as their own longitudinal controls, the absence of a comparator arm limits causal inference. Second, the relatively small sample size restricts the generalizability of these results and impedes the detection of rare adverse events. Additionally, differences in the severity of OLP, such as reticular and erosive types, or the timing of remission may lead to variations in the analgesic effect of ibuprofen gargles. Our sample size limits the statistical power for subgroup analyses, and these findings should be considered exploratory. Similarly, although stratification of the LTE study according to the number of weeks after study initiation was suggested, the limited number of participants in the LTE study did not allow for meaningful week-based stratified analyses. Third, concomitant therapies for oral lesions were permitted, which reflects real-world practice. At baseline, 4 of 18 patients used concomitant topical medications for oral lesions **(**Table 1**)**. As shown in the Results, paired evaluation among patients with available data at both baseline and the end of the LTE period indicated no initiation or discontinuation of concomitant topical medications during follow-up (initiation 0, discontinuation 0; exact McNemar test, *p* = 1.00), and no concomitant systemic analgesics were recorded during the LTE study. Nevertheless, concomitant therapies may still have influenced the observed clinical course, and the treatment effect of ibuprofen gargling should be interpreted with caution. Fourth, we analyzed multiple PROMS domains simultaneously without adjusting for multiple comparisons, thereby increasing the risk of Type I errors. Therefore, the further application of appropriate statistical corrections may render some of our significant findings non-significant, particularly for those with *p*-values close to 0.05. Fifth, although we demonstrated significant improvements in various PROMS scores, the improvements are gradual and modest and suggesting that ibuprofen gargle had little time-dependent effects. The clinical meaningfulness of these changes remains uncertain because of the absence of established minimal clinically important difference thresholds for the PROMS domains in patients with OLP. The estimated time-dependent slopes (β_day_) were generally modest across PROMS domains. Additionally, the ICCs ranged from approximately 0.2 to 0.8, suggesting that some domains were dominated by between-subject differences whereas others showed substantial within-subject day-to-day variability. This heterogeneity in variability structure should be taken into account when interpreting the magnitude and precision of the estimated slopes. The observed daily improvements, though significant, may not represent the perceptible benefits for individual patients. Future research should be conducted to validate these findings in larger multicenter randomized controlled trials. In addition, comparative studies against established therapies, such as topical corticosteroids, are required to define the relative efficacy of ibuprofen gargling and explore any potential synergistic effects in combination regimens. Moreover, pharmacokinetic and mechanistic studies would further elucidate the extent of systemic exposure and the pathways that underlie the observed functional improvements. Finally, research identifying patient subgroups that would most likely benefit from this treatment, such as those with predominant pain during mastication, may enable more personalized treatment approaches.

## Conclusions

In conclusion, this exploratory study suggests that a six-month regimen of ibuprofen gargling may be safe and associated with modest and gradual improvements in pain and oral function-related QOL in patients with OLP. However, these findings should be interpreted with caution, and further confirmatory study is needed to confirm the potential therapeutic role of ibuprofen gargling.

## Data Availability

The datasets used and/or analyzed during the current study are available from the corresponding author upon reasonable request.

## Abbreviations

CTCAE: The Common Terminology Criteria for Adverse Events
ICC: Intraclass correlation coefficient
LMMs: Linear mixed-effects models
LTE: Long-term extension
NSAIDs: Nonsteroidal anti-inflammatory drugs
OLP: Oral lichen planus
PROMS: Patient-reported oral mucositis symptom
SD: Standard Deviation
QOL: Quality of life
VAS: Visual analogue scale
